# A qualitative exploration of how lifetime stressor exposure influences sport performers’ health, well-being, and performance

**DOI:** 10.1080/10615806.2023.2246023

**Published:** 2023-09-04

**Authors:** Ella McLoughlin, Rachel Arnold, Lee J. Moore, George M. Slavich, David Fletcher

**Affiliations:** a Department for Health, University of Bath, Bath, UK;; b School of Science and Technology, Nottingham Trent University, Nottingham, UK;; c Department of Psychiatry and Biobehavioral Sciences, University of California, Los Angeles, CA, USA;; d School of Sport, Exercise and Health Sciences, Loughborough University, Loughborough, UK

**Keywords:** Adversity, assessment, life stress, qualitative, timelining

## Abstract

**Background and objectives:**

Recent research has shown that lifetime stressor exposure can negatively impact sport performers. However, this work has predominantly relied on quantitative methods, which has provided limited information regarding *how* stressors occurring over the life course affect health, well-being, and performance. This study aimed to explore how relatively high levels of lifetime (non-sport and sport-specific) stressor exposure influenced sport performers’ health, well-being, and performance.

**Methods and Design:**

To identify participants who had experienced high lifetime (non-sport and sport-specific) stressors, we used criterion-based purposeful sampling from a prior study. Subsequently, semi-structured interviews, complemented by timelining, were conducted with 22 sport performers (17 female; *M*_*age*_ = 25.89, *SD* = 10.20).

**Results:**

We used reflexive thematic analysis to develop three overarching themes that illustrate how high lifetime (non-sport and sport-specific) stressor exposure influences sport performers’ health, well-being, and performance. These were: psychological (e.g., maladaptive coping strategies), social (e.g., difficulties in building relationships), and behavioral (e.g., risky behaviors) factors.

**Conclusions:**

These findings can help practitioners identify sport performers at risk of developing stress-related health, well-being, and performance problems, and may aid the development of effective interventions.

The experience of psychological stress is idiosyncratic with the sporting environment, whereby sport performers are exposed to various stressors ranging from mundane daily hassles to major life events (Arnold & Fletcher, 2021). Research within the sporting domain has revealed that many of these stressors are associated with performing in a competitive sporting environment (e.g., underperformance), the sporting organization within which athletes operate (e.g., coach-athlete relationship), and personal non-sporting life events (e.g., death of a relative; Fletcher et al., 2006). Although engagement in sport can contribute to enhanced health and well-being (Rice et al., 2021), it is well-established that sporting involvement can negatively impact sport performers, due to the stressors they encounter during their careers (Poucher et al., 2019). As a result, there is compelling evidence to suggest that stressors are a salient feature of sport performers’ lives, which may degrade their health, well-being, and performance (Arnold & Fletcher, 2021; Giles et al., 2020; Gouttebarge et al., 2019; Lundqvist, 2021).

Despite the general appreciation of the negative effects that stressors can have on health, little research has explored the multidimensional nature of lifetime stressor exposure (Slavich, 2019), particularly among sport performers (c.f. McLoughlin et al., 2021). Specifically, in the sport psychology literature, research has predominantly focused on sport performers’ experience of a specific life event (e.g., injury), as opposed to understanding the combined and cumulative effect of sporting and non-sporting stressors over the entire lifespan (Fletcher, 2019). Lifetime stressor exposure refers to the combined and cumulative effect of stressors occurring over the entire lifespan, and includes acute life events (e.g., death of a loved one) and chronic difficulties (e.g., ongoing health problems; Mayer et al., 2019). This is an important area of enquiry considering that the accumulation of stressors is one of the conditions that leads to allostatic load and disease (McEwen, 1998). To elaborate, the exclusive focus on a single event undermines the significance of time and temporality within a sport performers’ life experiences (Mannay, 2021). As Howells and Fletcher (2015) remarked, it is incongruous to assume that a single stressor could be considered separate from the wider human experience. Rather, the presence of stressors (e.g., injury) frequently leads to others (e.g., financial problems), and can influence how sport performers respond to future stressors (Howells & Fletcher, 2015). Accordingly, the adoption of a more holistic view of stress could generate a more nuanced and complete understanding of sport performers’ experiences, capturing how their present thoughts and feelings are affected by past experiences (Mannay, 2021).

One theoretical framework that explains how stress affects health is the integrative model of lifespan stress and health (Epel et al., 2018). Specifically, this model has three elements: (1) contextual factors, including individual and environmental factors (e.g., genetics and developmental contexts), cumulative stressor exposure (e.g., historical and current stressor exposure), and protective factors (e.g., family support); (2) psychophysiological stress responses (e.g., cardiovascular reactivity); and (3) biological aging and disease (e.g., cardiovascular disease). Broadly, this model suggests that greater lifetime stressor exposure alters how individuals interpret (e.g., greater threat appraisals) and respond to (e.g., heightened psychophysiological responses such as state anxiety and heart rate) stressors, which can in turn negatively affect health (Epel et al., 2018). As a result, the integrative model aims to explain the potential mechanisms linking stressors and health (Epel et al., 2018).

Recently, lifetime stressor exposure has been examined in relation to various outcomes (e.g., Pegg et al., 2019), with studies generally showing that greater lifetime stressor exposure is associated with poorer mental and physical health (Slavich & Shields, 2018). Importantly, the negative association between lifetime stressor exposure and health holds for both the frequency of stressors encountered and their severity (Slavich & Shields, 2018). Indeed, greater lifetime stressor exposure, both in terms of frequency and severity, has been related to greater symptoms of depression (Pegg et al., 2019) and anxiety (Slavich et al., 2019), as well as poorer physical health (Cazassa et al., 2020). However, some studies have found lifetime stressor severity to be a stronger predictor of ill-health than the total number of stressors experienced (Slavich et al., 2019).

To further extend this literature, researchers have examined the potential mechanisms underlying the stressor-health relationship (Lam et al., 2019; Mayer et al., 2019). For example, research has examined underling biological mechanisms, finding that greater lifetime stressor exposure was associated with blunted cortisol and heightened DHEA reactivity to an acute laboratory-based stressor (Lam et al., 2019). Similarly, greater lifetime stressor exposure has been associated with shorter leukocyte telomere length (Mayer et al., 2019). These results reveal that lifetime stressor exposure may initiate several stress-related changes in hormonal activity and telomere length, which could lead to poorer health outcomes (e.g., cardiovascular disease; Mayer et al., 2019). Research has also examined underlying neural processes, revealing that greater lifetime stressor exposure was associated with reduced social reward responsiveness, resulting in increased vulnerability to depression (Pegg et al., 2019). Finally, research has investigated underlying psychological mechanisms, revealing that greater emotion-focused coping (i.e., forgiveness) buffered the negative effects of lifetime stress on mental health (Toussaint et al., 2016). This research indicates that the lifetime stressor exposure-health relationship can be partly explained through biological, neural, and psychological processes (e.g., Lam et al., 2019). Although this quantitative-based research has been insightful, combining this approach with qualitative methods may help researchers further understand *how* exposure to lifetime stressor affects health (Greene et al., 1989). Despite these advances, the association between lifetime stressor exposure and other outcomes, such as well-being and performance, during potentially stressful situations has not been investigated.

Initial research within a sporting context suggested that elite athletes who have experienced greater lifetime (non-sport) stressors exhibited more symptoms of depression and anxiety, and poorer psychological well-being (McLoughlin et al., 2021). Specifically, exposure to stressors that were chronic (vs. acute), or that have occurred in adulthood (vs. early life), were found to be particularly harmful to mental health and well-being (McLoughlin et al., 2021). More recently, these results were extended by McLoughlin et al. (2022), who found that greater lifetime (non-sport and sport-specific) stressor exposure was associated with mental and physical ill-health (e.g., depression). Although greater lifetime (non-sport) stressor exposure was significantly associated with lower well-being, these results were not consistent in relation to lifetime (sport-specific) stressor exposure. Given the mixed findings reported between lifetime stressor exposure and well-being, qualitative methods could help unpick these equivocal findings by revealing the subtleties and complexities of the variables being studied (Anderson, 2010). The results also revealed that sport performers who experienced more severe lifetime (non-sport and sport-specific) stressors were more likely to habitually appraise potentially stressful events as threatening (McLoughlin et al., 2022). Although this research provides initial insights into the potential mechanisms underpinning the stressor-health effect, qualitative methods could provide an even deeper understanding of sport performers’ experiences of high lifetime (non-sport and sport-specific) stressor exposure and the factors they believe link such exposure to key outcomes (e.g., health, well-being, performance).

Moreover, despite some studies exploring sport performers’ experiences of stressors over extended periods of time (e.g., Galli & Reel, 2012; Howells & Fletcher, 2016), to our knowledge, only one study has used qualitative methods to explore the subjective perceptions of sport performers who have experienced high lifetime (non-sport and sport-specific) stressor exposure (McLoughlin et al., 2021). Specifically, this study used mixed-methods to explore the links between lifetime stressor exposure, mental health, and well-being in elite athletes. Despite being a primarily quantitative study, follow-up interviews with six athletes demonstrated that greater lifetime (non-sport) stressor exposure fostered poorer mental health and well-being by promoting maladaptive long-term coping strategies, increasing susceptibility to future stressors, and limiting interpersonal relationships (McLoughlin et al., 2021). However, the qualitative data obtained were limited given that only non-sport stressors were examined, and the sample was restricted to elite athletes. Given that stressors are encountered by sport performers competing at various standards (e.g., amateur to elite), future research should extend these findings among sport performers from a range of competitive levels (vs. elite-athletes only; Arnold et al., 2017a, 2017b).

Therefore, the present study extended past research by providing a more in-depth understanding of lifetime (non-sport and sport-specific) stressor exposure with sport performers from multiple competitive level (club to senior international). Specifically, this study aimed to explore how relatively high levels of lifetime (non-sport and sport-specific) stressor exposure influenced sport performers’ health, well-being, and performance.

## Method

### Research design

This research was underpinned by ontological relativism (i.e., reality is multiple, created, and mind-dependent) and epistemological constructionism (i.e., knowledge is constructed and subjective). Consistent with these assumptions, we conducted semi-structured interviews and analysed the data using reflexive thematic analysis (Braun & Clarke, 2019a). Within this paradigm, there is an understanding that there is no social reality independent of us that can be accessed and that realities are subjective, multiple, created, mind-dependent, and change over time (Smith & Sparkes, 2009). It is important to note here that the accounts we present in the findings are thus based on the first authors’ interpretations and contextualized within her own experiences as a 26-year-old white woman from a working-class background and understanding that this topic is one that she is personally invested in due to her own personal experiences with stressors. To elaborate, the interviewer (i.e., the first author) has conducted over 50 in-depth interviews with a range of participants (e.g., elite athletes, student-athletes), discussing sensitive topics (e.g., abuse). These prior experiences enabled the first author to better prepare for such conversations as a researcher, connect with participants on a deeper level, and listen to their stories with greater empathy and curiosity.

### Participants

We identified participants who had experienced high lifetime (non-sport and sport-specific) stressor exposure via criterion-based purposeful sampling. This strategy was used on participants drawn from a prior study of 395 sport performers (*M*_*age*_ = 22.50 years, *SD*_*age*_ = 5.33; McLoughlin et al., 2022). We identified participants in the top 10% of lifetime stressor count (*M* = 30.33, *SD* = 10.93, Range = 18–65) or severity (*M* = 67.81, *SD* = 21.36, Range = 32–111), as assessed by the Stress and Adversity Inventory for Adults (Slavich & Shields, 2018) and the Sport Stress Assessment Module (McLoughlin et al., 2022). Following the removal of duplicates across the top 10% of lifetime (non-sport and sport-specific) stressor count or severity, a total of 64 participants were invited to take part via email. Given the response rates documented in prior research (e.g., 16.5% in Sappleton & Lourenço, 2016), this participant pool was deemed appropriate to provide a manageable sample size. Furthermore, such an approach is consistent with prior research in sport psychology (e.g., Barnes et al., 2021). This was deemed as high lifetime stressor exposure when compared to the total count (*M* = 14.00, *SD* = 11.91) and severity (*M* = 28.00, *SD* = 25.63) of lifetime (non-sport and sport-specific) stressors experienced in McLoughlin et al. (2022). In total, we interviewed 22 sport performers (17 female, 5 male; *M*_*age*_ = 25.89 years, *SD* = 10.20).

Participants were from a range of individual (e.g., badminton) and team (e.g., hockey) sports and had an average of nine years’ experience competing in their sport (*SD* = 5.01). Participants performed at various competitive levels, including senior international (*n* = 5), international (*n* = 5), national (*n* = 4), regional (*n* = 5), university (*n* = 2), and club (*n* = 1). This sample provided sufficient information power given the quality of interview dialogue between the researcher and participants (Malterud et al., 2016). This is consistent with Braun and Clarke (2019b), who suggested that information power is a useful alternative to data saturation for thinking around justifications for sample size in reflexive thematic analysis. To elaborate, asking participants to complete the timelining activity prior to the semi-structured interview enabled a focused dialogue and aided rapport (Malterud et al., 2016). Finally, the homogeneity of the sample provided sufficient information power given that all participants had experienced the phenomena in question. The sample was deemed homogenous given that sport performers have been purposefully sampled for participation in this study whereby they had been exposed to a relatively high and/or severe amount of lifetime (non-sport and sport-specific) stressors, and they were all sport performers aged 18 or over.

### Data collection

Institutional ethical approval was granted from the Research Ethics Approval Committee for Health (REACH) at the University of Bath (EP 19/20 088). Participants who met the inclusion criteria (i.e., top 10% of lifetime [non-sport and sport-specific] stressor count or severity) were invited to participate (*n* = 64). Participants who were willing to be interviewed (*n* = 22; response rate = 34%) were re-contacted with study information and notified of their ethical rights (e.g., confidentiality, right to withdraw). Prior to the interviews, all participants provided informed consent. Consistent with recommendations made by Day and Wadey (2017) for collecting interview data around sensitive topics, we minimized the discomfort that may arise from revisiting and discussing potentially traumatic events by using multiple methods for data collection and spending an adequate amount of time with participants. Specifically, participant control is a vital element when exploring sensitive topics, as it encourages an equal relationship between interviewer and participant (Hanna, 2012). We facilitated this by giving participants control over when the interview was conducted, the order of the topics, and making the interviews participant-led (vs. researcher-led). The use of timelining also gave participants control over what stressors they were comfortable in discussing, and in what order (e.g., participants were not required to speak through their life story in chronological order; Day & Martinelli, 2016). After the interviews, all participants were signposted toward support services (e.g., Samaritans). Although the focus was on how best to protect participants, we also ensured that the interviewer (i.e., the first author) had sufficient support and de-brief opportunities via academic and counseling supervision. This debriefing occurred weekly and consisted of the first author detailing any reflective thoughts from the interviews and expressing any emotions that were incited throughout the data collection process. Interviews were conducted on Microsoft Teams given that data collection occurred during a period of national lockdown caused by the Coronavirus pandemic. The interviews lasted between 61 and 160 min (*M*_*duration*_ = 81.80 min; *SD* = 21.20), were recorded using a Dictaphone, and then transcribed by the first author.

To better capture participants exposure to lifetime stressors, and thus extending prior research, participants were asked to create a visual timeline of their life story, with an emphasis on the “highs and lows” they had experienced both outside and inside of sport. Prior to completing their visual timeline, participants were provided with “made-up” example timelines to help them construct their own timeline (see Supplementary Materials). Engaging in creative methods such as this encourages participants to spend more time reflecting on the topics under study, deepening their understanding of past experiences, and improving their ability to recall events (Mannay, 2021). Participants were posted materials to construct their timelines (e.g., colored pens, emotion stickers), which were then shared securely with the first author via email prior to the interviews. Semi-structured interviews were selected as they provided the opportunity to explore detailed accounts of participants’ experiences of lifetime (non-sport and sport-specific) stressors (Trainor & Bundon, 2021). When conducting the interviews, the first author used an interview guide to ensure key topics were covered. However, discussions were typically guided by what she interpreted to be important and meaningful. We developed a semi-structured interview guide, which comprised of four sections, including: (1) introductory questions relating to their sporting involvement (e.g., How did you first get involved in your sport?), (2) questions about participants’ exposure to stressful life events (e.g., Could you please tell me about the stressors you have experienced outside of sport?), (3) questions exploring the impact of stressors on participants’ health, well-being, and performance (e.g., Do you think these stressors had an impact on your mental health? If so, can you elaborate on this impact?), and (4) questions regarding participants’ responses to stressors (e.g., How do you typically respond to stressful events?).

### Data analysis

To identify patterns of meaning within the data, we used reflexive thematic analysis (Braun & Clarke, 2019a). Although presented sequentially, the progression through phases was not direct and instead involved a recursive process of moving forwards and backwards through data familiarization, coding, theme development, revision, naming, and writing-up (Braun & Clarke, 2022). Initially, the process involved the first author familiarizing herself with the data and making preliminary notes. Given that the first author led on every aspect of the data collection process (e.g., developing the interview guide, conducting and transcribing interviews), she was immersed in the data prior to formal analysis. During this phase, the first author re-familiarised herself with the data by listening to the audio recordings while simultaneously reading and re-reading the transcripts. At this point, she noted any initial trends in the data and identified any interesting passages using the highlighter function in Microsoft Word. A reflexive journal was used throughout to note down any personal reflections, thoughts, or feelings. The preliminary iteration of coding was conducted using the “comments” function in Microsoft Word, by identifying data that may be useful in answering the research question. Throughout this process, the first author double-coded the data in accordance with the semantic meaning communicated by participants, and the latent meaning she interpreted (Patton, 1990). The first author also played an active role in interpreting codes by identifying any hidden meaning within the data (Byrne, 2021). This was guided by a predominantly inductive approach, meaning that data was “open-coded” to best represent meaning as communicated by the participants (Braun & Clarke, 2022). However, a degree of deductive analysis was used to ensure that the open coding was relevant to the research question and aligned with the theoretical framework which underpinned this study (Byrne, 2021). To elaborate, the integrative model of lifespan stress and health highlights factors which may increase vulnerability and resiliency to stressors (e.g., supportive family structures, cognitive biases; Epel et al., 2018).

Once all of the data were coded, the focus shifted from the interpretation of individual data items to aggregating meaning and meaningfulness across the dataset (Byrne, 2021). This resulted in the initial list of codes being collated into a Microsoft Word document and organizing them in a way that reflected some commonality in what participants expressed. When developing initial themes, the first author manually organized codes by printing them off and clustering similar codes together, which then started to form candidate themes for each cluster (Trainor & Bundon, 2021). Additionally, this process involved collapsing multiple codes that shared an underlying concept. To elaborate, the initial codes of “procrastination” and “withdrawal” were merged into “avoidance” as they were highly synergistic and ultimately referred to avoiding stressful life events but via different means. In the next phase, the first author reviewed the potential themes to ensure a coherent and logical relationship between the data items and codes that inform each theme. It was during this phase that codes and themes were removed to facilitate the most meaningful interpretation of the data. To help facilitate this process, the first author created a thematic map to illustrate the relationships between themes. Finally, themes were defined and named before being written-up.

### Rigor and trustworthiness

Consistent with ontological relativism and epistemological constructivism, a flexible list of criteria was developed to enhance the quality of the data collected, including: the worthiness of the topic, rich rigor, the significant contribution of the work conducted, meaningful coherence, and methodological integrity (Levitt et al., 2018). First, the worthiness of the topic was established given that there is a gap in the literature regarding our understanding of *how* high lifetime (non-sport and sport-specific) stressor exposure influences sport performers’ health, well-being, and performance. Second, rich rigor was achieved by conducting pilot interviews, keeping a reflexive journal, and engaging in critical dialogue. Indeed, three pilot interviews were conducted to ensure interview questions and probes were appropriate, and highlighted that some questions were too ambiguous and needed rephrasing (e.g., “How do you typically evaluate the demands of a stressful situation?” became “How do you typically perceive stressful life events?”).

Reflexive journaling was also used to critically reflect upon prior assumptions and biases. Identifying the first author’s personal standpoints (e.g., as a 26-year-old white woman from a working-class background) and understanding that this topic is one that she is personally invested in, served to contextualize how her positionality influenced the research and data analysis process. Furthermore, the reflexive journal was used to acknowledge functional (i.e., how the methods and other aspects of design shape the research and knowledge produced) and disciplinary (i.e., how academic disciplines shape knowledge production) reflexivity (Braun & Clarke, 2022). For example, acknowledging that the first author was an outsider to the group she was researching and so understanding that participants may not feel comfortable disclosing certain events. As a result of these challenges, the first author dedicated a significant amount of time in building rapport with participants and conducting this research in an ethical and sensitive manner (Braun & Clarke, 2022). It is important to note that we consider subjectivity to be a resource when collecting and analysing data (Gough & Madill, 2012). Indeed, this subjectivity allowed the first author to connect on a deeper level with participants, listening to their stories with empathy and genuine curiosity. Third, throughout the data analysis process, the second and third authors served as “critical friends,” challenging my construction of knowledge. For example, the second and third author questioned the initial code of “self-harm” and asked why this only included supporting quotes which referred to participants physically harming themselves. As a result, this was subsequently reworded into “self-injury” to make this more aligned to the supporting quotes. These “critical friends” provided a useful check-in for the limits of reflexivity, whereby co-authors questioned things that the first author may not have considered (Braun & Clarke, 2022). Finally, the study sought meaningful coherence and methodological integrity by considering how the study aims, approach to enquiry, design and procedure, and findings, all fitted together (Levitt et al., 2017).

## Results

We developed three overarching themes relating to the psychological, social, and behavioral factors that may provide some insight into *how* lifetime (non-sport and sport-specific) stressors influence sport performers’ health, well-being, and performance. Each overarching theme is introduced in the following narrative, with its themes elaborated on and accompanied by illustrative quotes. Parts of these quotes are italicized where participants indicate *how* lifetime stressor exposure influenced their health, well-being, and performance.

### Psychological factors

This overarching theme that we developed encompassed three themes relating to the psychological factors that may provide some insight into *how* lifetime (non-sport and sport-specific) stressor exposure influences health, well-being, and performance, including: maladaptive coping strategies, irrational thoughts and beliefs, and dysfunctional emotional regulation strategies.

#### Maladaptive coping strategies

Many participants reported using maladaptive coping strategies to deal with the stressors they experienced. These maladaptive coping strategies can be defined as the cognitive strategies employed to manage these stressors. First, several participants explained how denial was used as a maladaptive coping strategy to deal with stressors. Specifically, denial was characterized as blocking events from conscious awareness by refusing to acknowledge the reality of a stressor. The following quote exemplifies denial being used as a maladaptive coping strategy by one sport performer:

A boy invited me to his house to teach me how to play guitar and in my head, I was always going to play guitar with him … But he did a very, very, very horrible thing to me when I said no. As a 13-year-old girl, *I was never expecting that in a million years … The fact that I didn’t even write it down on the timeline is proof that I still haven’t accepted what happened*.

(Participant 16)

The use of denial over a prolonged period could potentially prevent cognitive processing of stressors and may have exacerbated the impact lifetime stressors can have on sport performers’ health and well-being. This was illustrated by the following quote:

My parents divorced when I was 8 years old because my dad was verbally and physically abusive … *It never really affected me at the time. I had blocked off a lot of things that happened during my childhood and I forgot about it*. It was when flickers of memories came back, and my mood then became very low, and I was very unhappy. Everything started to deteriorate from that point … There has been a lot of periods of depression throughout the last 10 years.

(Participant 5)

Some participants noted how they engaged in a process of self-blame following exposure to lifetime stressors. Self-blame was characterized by participants holding themselves responsible for causing or contributing to the stressors. This was illustrated by the following quote:

There was the situation where the guy followed me home and raped me, there was that situation in high school where I went to that boy’s house and he did bad things to me when I said no. There was another situation where this boy emotionally blackmailed me to get a photograph of me … *I felt like I put myself in those situations. A lot of situations that have happened, I feel like they are my fault*.

(Participant 16)

Additionally, the use of self-blame could have promoted negative attributions toward participants’ own behaviour, potentially resulting in mental health problems (e.g., anxiety disorders). This was exemplified by the following quote:

My boyfriend physically assaulted me, and *I very much thought that I brought it on myself*. I thought that I was everything that he was using to describe me. I thought he was right … I thought that I was too fat, I thought that I wasn’t like everyone else and I wasn’t like those other girls … *I blamed myself for what happened*. That was when everything went really, really badly, really, really quickly … That was when my eating behaviours started to change, and my anxiety really started to pick up.

(Participant 8)

Finally, several participants noted how they engaged in avoidance coping to potentially delay dealing with stressors. This was illustrated by the following quote:

I would always just try to avoid it and shove it into a box or something. When I was injured for example, *I threw myself into coaching and work a lot more* … Similarly, my mum struggles with alcohol addiction and if she was ever in a state, *I always had the excuse that I had netball coaching … I tend to busy myself with other things to avoid actually dealing with them*.

(Participant 6)

As a result, avoidance coping strategies such as procrastination could lead to intrusive thoughts and heightened negative feelings. This was shown by the following quote:

As soon as a rugby ball was kicked, it was a good way to get away from things and a place where you don’t really concentrate on things. I think the hardest thing for me is when that happens, you forget about everything and then *the moment you finish, everything just floods back, and it hits you like a tonne of bricks*.

(Participant 21)

#### Irrational thoughts and beliefs

Some participants engaged in irrational thought patterns after experiencing a high number of stressors. These thought patterns were characterized by inaccurate beliefs that were used to reinforce negative thinking, and included: minimizing, over-generalization, polarized thinking, catastrophizing, and fallacy of fairness. To elaborate, minimizing involved reducing the importance and significance of stressors, as shown by the following quote:

My parents divorced in the early 90’s and after that there was an incident with my mum’s partner. He was quite violent, and we had to hide from him. I suppose, *I didn’t write that on the timeline because it doesn’t seem that important to me*.

(Participant 3)

As a result, minimizing stressful life events could have detrimental consequences on sport performers’ mental health, as illustrated by the following quote:

I thought I dealt with it [sexual assault] really well because *I said that it wasn’t as significant as first thought*. That was very effective in the short-term, but in the long-term that had a massive effect on my mental health.

(Participant 16)

Most participants also engaged in catastrophizing, which occurs when someone assumes the worst-case scenario. This was demonstrated by the following quote:

I am over-protective towards my two younger children, even the simple thing like today, they might be going in the same car together and that immediately makes me think, I don’t want them in the same car. *What happens if they skid on the ice and were both killed?* … I think that is directly from having a child who is profoundly disabled, and from the feeling that your children are very, very vulnerable.

(Participant 17)

Some participants also engaged in an unhealthy thinking pattern characterized by rigid, absolute, and unchangeable beliefs. This was illustrated by the following quote:

When I am stressed, *I try to resolve things through excessive planning and organization and doing things a certain way. I will have to do a certain number of laps or a certain number of stretches* … It is kind of obsessive. *If I don’t do it then I won’t be the best I can be*.

(Participant 5)

As a result, mental rigidity could have deleterious consequences on mental health, as these beliefs are often illogical, false, and unhelpful. This was exemplified by the following quote:

I tried to control things in my life because I couldn’t seem to control anything else. I just cleaned and cleaned and cleaned … I couldn’t stop. *I became almost obsessed and blinkered, which led to other issues. I went into a bit of a hole, and I found it really difficult to get out of that cycle*.

(Participant 9)

#### Dysfunctional emotional regulation strategies

First, many participants disclosed that they engaged in dysfunctional emotional regulation strategies to make uncomfortable emotions (e.g., sadness, anger) more manageable. Specifically, emotional suppression involved consciously inhibiting the overt expression of emotion. In support of this, one participant stated:

When my mum was diagnosed with breast cancer, I cried when she told me, but I haven’t cried since. Everyone was always asking me if I was upset, and I wasn’t. *I don’t feel as if I show emotions externally. I am not one of those people who cry at everything, I just don’t*.

(Participant 16)

Additionally, these participants also explained how suppressing intense emotions in childhood could lead to emotional repression in adulthood because of primary caregivers invalidating their expression of emotion. This was illustrated by the following quote:

My relationship with my dad is weird. When I was really young, he would shout a lot and I used to cry because he was shouting at me. That used to make him angrier, so through that *I learnt not to cry. If I was going to cry, I could just hold it in. I just then found because I wasn’t crying, I just wasn’t getting emotional anymore*. All of the other times when family members have died, I just haven’t cried. I didn’t cry at my Grandad’s funeral, I didn’t cry at my cousin’s funeral … I haven’t cried at things like that. It is just something that I have learnt not to do.

(Participant 8)

Participants also mentioned displaying extreme emotional outbursts following exposure to stressors, which were characterized by intense and exaggerated emotions (e.g., sadness, anger). This was illustrated by the following quote:

My mum and dad split up because my dad was having an affair … I wasn’t enjoying the job I was in. I had an injury, so I had stopped playing netball … *I remember I just burst out crying in the office one day and I have never done that in my life, and I am not like that as a person. I don’t tend to get upset easily, but I think I just had to explode in some way*.

(Participant 6)

It was evident in our analysis that inhibiting the expression of emotions led to emotional outbursts, which could have negative consequences for health and well-being. This was supported by the following quote:

*I would be feeling all of this stuff, but not expressing any of it … I would break down in tears just as a way to release of all that pent-up emotion*. That would always induce some sort of panic attack just to try to cope with everything.

(Participant 8)

Finally, several participants noted that they engaged in rumination following exposure to lifetime stressors. Rumination was portrayed as a tendency to repeatedly focus on negative events and the thoughts that followed. This was illustrated by the following quote:

I was playing a [rugby] game and I came round the corner and there was a space outside the defender. I was lazy and didn’t get round the corner in time. *For three weeks after, I would think about that game constantly and just ask why didn’t I run 20% faster, why didn’t I do that? If I had done that, I might have scored a try and won the game*.

(Participant 21)

The process of rumination or dwelling on past failures can result in “paralysis,” where sport performers become so fixated on an event, they become unable to respond in a productive way. This was supported by the following quote:

I would overthink … Instead of actually doing something, I would be thinking about all these different things. *I would be stressing so much about things, that I actually wouldn’t do anything. I spent so much time worrying about things, I just never got anything done* … It definitely had an impact on my mental health and it just got to the point where I just thought it was too much. If my mum didn’t stop me, I would have just ended it right there.

(Participant 11)

### Social factors

This overarching theme encompassed two themes relating to the social factors that may provide some insight into *how* lifetime (non-sport and sport-specific) stressor exposure influences health, well-being, and performance, including: difficulties in building and maintaining relationships, and future relationship dysfunctions.

#### Difficulties in building and maintaining relationships

Most participants experienced difficulty in building and maintaining relationships because of experiencing high lifetime stressors. The following quote illuminates how previous stressors can leave an indelible impression on an individual’s ability to trust others:

I was invited to the Commonwealth Games, which was just a dream come true. But in the last warm-up day before the competition started, I slipped and tore the cartilage in my knee. I was on crutches for 8-weeks … And then, my boyfriend cheated on me. I was head over heels in love. He was just the best and he cheated on me and it was just like, I thought that the relationship was good … From a boyfriend point of view*, I don’t trust people so easily. I don’t trust that if something bad happens again, that they are always going to be there*.

(Participant 9)

Furthermore, most participants noted fragmented relationships with others due to the difficulty in confiding with those who had not encountered similar stressors in their lives. This notion was supported by the following quote:

*I wanted someone to relate to, I wanted someone to say they have had those experiences*. That is one of my biggest frustrations … I have spoken to my partner’s mum in the past and she has tried to link things to her life, and it is completely just not comparable, and it just infuriates me because *I’m like you don’t understand*.

(Participant 6)

Finally, several participants explained how high lifetime stressor exposure led to decreased communication with others to conceal feelings of fragility and vulnerability. This was demonstrated by the following quote:

My dad was diagnosed with terminal cancer and one of my friends at school died of suicide … In the 3-month period where all that happened, *I didn’t talk to anyone about it. My teachers weren’t aware of it, my friends weren’t aware of it*. No-one was aware of what was going on because I was determined to keep it all separate from them. I thought showing that emotion was some kind of weakness … It just kind of snowballed and it affected my mental health very poorly.

(Participant 1)

Consequently, participants viewed vulnerability and strength as mutually exclusive constructs, attempting to emanate a distorted image of “mental toughness” to others.

#### Future relationship dysfunctions

Most participants noted that exposure to high lifetime stressors inhibited the formation of intimate relationships. This was characterized as a deep-seated fear of close emotional or physical relationships with others, as illustrated by the following quote:

The whole situation with my dad having an affair really highlighted to me the fragility of relationships. Even relationships that you think are solid, my parents have been together for 30 years. *It has genuinely made me terrified of getting into a relationship with someone because I know it doesn’t take a lot for someone to turn around the next day and realise that they don’t want to be with you anymore*. That is terrifying.

(Participant 22)

Therefore, these accounts demonstrated that certain stressors (e.g., parental infidelity) may lead to a paralyzing fear of intimacy and abandonment by significant others.

Some participants also explained how exposure to high stressors over the lifetime led to the adoption of unhealthy relationships which were characterized by insecurity, dominance, and control. This was illustrated by the following quote:

With my most recent relationship, that was built on the biggest stress [sexual assault] that I have ever had to deal with. *That relationship went from naught to a hundred really quickly because he was the one person that I could confide in. I had this anxiety that something bad might happen again, so our relationship was very intense … Everything was happy but then it got very abusive and manipulative, and he made sure that I was his and no-one else’s*.

(Participant 16)

Furthermore, this participant noted how unhealthy relationships could have harmful consequences for their physical health:

My whole body was shutting down because of the stress caused from everything that happened. *I had really bad periods, I had 3 months straight of bleeding*. To the point where they did blood tests, and I was releasing the same hormones as you release when you go into labour. *My physical health was just down the drain*.

(Participant 16)

### Behavioral factors

This overarching theme encompassed various risky behaviors that may provide some insight into *how* lifetime (non-sport and sport-specific) stressor exposure influences health, well-being, and performance.

#### Engagement in risky behaviors

Following exposure to high lifetime stressors, several participants disclosed that they had engaged in risky behaviors. This was illustrated by the following quote:

When I was at school, I was an absolute pain. I was suspended all of the time. *I really channelled the stress I was under [parent diagnosed with terminal illness] into being disruptive … I got suspended twice for smoking on campus*. It was stupid but I just didn’t care. It was just something to make me feel something else other than sad, angry, or upset about what was going on in my life.

(Participant 1)

Although engagement in risky behaviors sought to provide a temporary relief, these behaviors sought to intensify negative feelings and have a detrimental impact on mental health and well-being in the long-term. This was illustrated by the following quote:

There was a period for around three to four months where I was just drinking constantly. I was doing it for fun with my friends, but I also knew that *it was just masking how I was really feeling. I didn’t realise how bad it got and it resulted in a suicide attempt*.

(Participant 5)

Additionally, several participants noted that they engaged in maladaptive eating behaviors, which could potentially be due to a lack of effective coping strategies that are important for successful functioning. This was supported by the following quote:

Throughout school, I was bullied … My eating disorder became my coping mechanism, and it was detrimental to my health. *More games than I care to admit, I would be sick beforehand, I would be sick during, I would be sick afterwards … I would make myself sick just to try and help me cope*.

(Participant 8)

This notion was further supported by another participant who engaged in self-injury throughout adolescence, potentially due to an absence of other effective coping strategies:

When I was younger, my dad was involved in drugs, so he wasn’t really there and when I did see him, he wasn’t very nice. I also got bullied in my first secondary school and I didn’t have a good time there. It was around that age where there was a stumble in my mental health … *When I was around 12, I self-harmed quite a lot and I used to cut myself, that continued until I was 15* … I don’t think there was a coping strategy, I think they were non-existent.

(Participant 5)

Therefore, experiencing high lifetime stressors led to greater engagement in risky behaviors, which might have negatively impacted health and well-being.

## Discussion

Greater lifetime stressor exposure has been associated with poorer health, well-being, and sports performance (e.g., McLoughlin et al., 2021). However, these findings are based primarily on quantitative methods and has largely failed to explore the factors that might help explain *how* lifetime (non-sport and sport-specific) stressor exposure influences sport performers’ health, well-being, and performance. This study aimed to explore how relatively high levels of lifetime (non-sport and sport-specific) stressor exposure influenced sport performers’ health, well-being, and performance. We used reflexive thematic analysis to develop three overarching themes. These were: psychological (e.g., maladaptive coping strategies), social (e.g., difficulties in building relationships), and behavioral (e.g., risky behaviors) factors. These themes support the integrative model of lifespan stress and health (Epel et al., 2018), which broadly suggest that greater lifetime stressor exposure negatively impacts health by influencing how individuals typically respond to stressful situations. The findings advance such theoretical understanding by providing an in-depth, original picture of sport performers’ psychological, social, and behavioral factors that link high lifetime (non-sport and sport-specific) stressor exposure to poorer health, well-being, and performance. As a result, these novel findings have the potential to help practitioners identify, and intervene accordingly with, those most at-risk for stress-related health and performance problems.

We developed three themes relating to the psychological factors, that may provide some insight into *how* lifetime (non-sport and sport-specific) stressor exposure influences health, well-being, and performance; namely, maladaptive coping strategies, irrational thoughts and beliefs, and dysfunctional emotional regulation strategies. First, participants reported using maladaptive coping strategies such as denial, self-blame, and avoidance to cope with stressful life events (e.g., parental divorce). This is consistent with extant literature (Fletcher et al., 2006), which also suggests that engagement in prolonged maladaptive coping strategies can induce a chronic and debilitating form of strain, which can result in sub-optimal mental health and well-being. Despite this, however, the effectiveness of certain coping strategies in preventing harmful consequences is complex (Madigan et al., 2020). For example, previous research has found that strategies such as denial can be useful in the short-term by minimizing distress and facilitating coping (e.g., Breznitz, 1983). However, in our data, such coping strategies were maladaptive, as they delayed the process of dealing with stressors, which maximized distress and impeded coping in the long-term. Thus, our study is the first to show that experiencing a high degree of lifetime stress might lead sport performers to use coping strategies that are ultimately maladaptive for their future health, well-being, and performance.

Some participants also disclosed that they had experienced irrational thoughts and beliefs following stressor exposure. Specifically, irrational beliefs can be defined as an unhealthy pattern of thinking that consists of rigid, extreme, and illogical beliefs (Turner, 2016). Indeed, irrational beliefs have been positively associated with various mental and physical health disorders (e.g., depression; Nelson, 1977). Moreover, the presence of irrational beliefs have also been linked to cognitive distortions such as catastrophizing, which has been found to predict mental ill-health among sport performers (e.g., Schnur et al., 2010). One potential explanation for this finding is that catastrophizing leads to poorer mental health via heightened anticipation of stressful life events (Sullivan et al., 2001). As a result, the findings of the present study provide initial, original evidence of how sport performers’ irrational beliefs may be developed (i.e., via high lifetime stressor exposure), which can inform interventions that protect their health (e.g., rational emotive behaviour therapy; Jordana et al., 2020).

Many participants noted how exposure to lifetime (non-sport and sport-specific) stressors led to the development of dysfunctional emotional regulation strategies (e.g., suppression). This corroborates the findings of prior research (e.g., Compare et al., 2014), wherein dysfunctional emotional regulation strategies have been implicated in the onset, maintenance, and exacerbation of mental ill-health. One potential explanation for this finding is that such strategies (e.g., rumination) enhance the intensity and valence of emotions (Calkins & Hill, 2007). To elaborate, prior research has found that intrusive ruminations (e.g., repetitive, negative, and unwanted thoughts) are associated with an inability to deal with stressful experiences (Tedeschi & Calhoun, 2004) and greater distress (Triplett et al., 2012). As a result, these findings advance prior research by implicating lifetime (non-sport and sport-specific) stressor exposure in the development of dysfunctional emotional regulation strategies, which participants subsequently perceive to contribute to poor health and well-being.

We developed two themes relating to the social factors that may provide some insight into *how* lifetime (non-sport and sport-specific) stressor exposure influences health, well-being, and performance; namely, difficulties in building and maintaining relationships and future relationship dysfunctions. It was clear that sport performers had difficulty in building and maintaining relationships because of the stressors they had encountered. According to interpersonal theories of depression (Hammen et al., 2014), exposure to stressors may initiate the development of interpersonal vulnerabilities (e.g., social withdrawal), which can lead to poorer health and well-being through psychophysiological pathways (Slavich & Irwin, 2014). Additionally, we found that support providers who had not had similar experiences to those faced by sport performers offered ineffective support, potentially due to a lack of mutual understanding. Therefore, the effectiveness of social support is highly dependent on the knowledge and experiences of providers (for a review, see Freeman, 2021). As such, it may be beneficial for support providers to possess high levels of contextual intelligence, as well as an awareness of the stressors faced by sport performers (Knight et al., 2018).

Furthermore, participants also stated that high exposure to lifetime (non-sport and sport-specific) stressors encouraged the formation of unhealthy relationships. Although social support has been frequently reported to buffer stress (Freeman, 2021), the present study revealed that relationships that have an unhealthy level of dependency may exacerbate the impact of stressors on sport performers’ health and well-being (Hayward et al., 2017). This is consistent with extant literature (e.g., Hammen et al., 2014), which suggests that stressor exposure could lead to mental and physical ill-health by encouraging dysfunctional social behaviors (e.g., pervasive dependence on others) and negative perceptions of relationships (e.g., subordination of one’s own needs). As a result, these findings support the “reverse-buffering effect” of social support, where ineffective or deficient support can intensify, rather than alleviate, the negative effects of stress (Arnold et al., 2018a). Despite the popularity of the stress-buffering hypothesis, future research should explore the “dark side” of social support (Ryff & Singer, 2000; see also Arnold et al., 2018b).

We developed one theme relating to the behavioral factors that may provide some insight into *how* lifetime (non-sport and sport-specific) stressor exposure influences health, well-being, and performance. Specifically, the present study found that many sport performers who experienced high lifetime (non-sport and sport-specific) stressors engaged in risky behaviors (e.g., excessive alcohol consumption). This supports prior research which has found that greater lifetime stressor exposure is associated with more risky behaviors (Slavich et al., 2019), possibly owing to the temporary relief they provide (Auerbach et al., 2007). Despite this short-term benefit, however, such behaviors may perpetuate the initial stressful circumstance (Auerbach et al., 2007). Therefore, our results add unique empirical evidence that high lifetime (non-sport and sport-specific) stressor exposure could play a vital role in the onset and maintenance of maladaptive behaviors which are ultimately detrimental for the health, well-being, and performance of sport performers (El Ghoch et al., 2013). Given that coaches play a key role in identifying concerns and facilitating help-seeking behaviors among athletes (Mazzer & Rickwood, 2015), our study provides accessible information to assist coaches in recognizing behavioral pre-cursors of mental ill-health (Biggin et al., 2017).

The findings of this study have some important theoretical and applied implications. In addition to supporting the integrative model of lifespan stress and health (Epel et al., 2018), our findings extend this model in various ways. First, although the model notes certain factors that shape vulnerability or resiliency to stress (e.g., self-esteem, physical activity), it only provides a relatively limited list of factors. Therefore, our novel findings add further insight and a more in-depth understanding into these factors and the complexities within them (e.g., benefits and drawbacks of social support). Furthermore, this study extended the propositions of this model beyond health by including well-being and performance as an outcome. However, the data garnered seemed to provide comparatively limited information on the factors that participants perceived to link high lifetime (non-sport and sport-specific) stressor exposure with their sporting performance. One potential explanation for the comparatively limited information linking lifetime stressor exposure to sporting performance may be due to the wide range of competitive levels among participants in the sample (e.g., club to senior international). Despite this, however, these findings demonstrate that the way in which participants respond to stressors may have an indirect effect on sporting performance. For example, sport represented an outlet for sport performers to delay dealing with stressors and to demonstrate obsessive behaviors. Such findings suggest that although stressor exposure may not impact performance per se, the way in which participants responded to lifetime stressor exposure may initially be deemed effective but may be linked to more maladaptive coping longer term.

Turning to the applied implications, our data suggest that practitioners should be aware of the competing demands placed on sport performers by extending traditional assessments of stressors experienced recently, to also considering their historical exposure to lifetime (non-sport and sport-specific) stressors. As a result, this will help identify those at-risk of developing stress-related health and performance problems. Relating this to practice, it likely means that sport psychologists will need to establish on-going working alliances with clinical psychologists, in an effort to protect sport performers’ health, well-being, and performance (Sly et al., 2020). Indeed, while sport psychology is a field primarily concerned with addressing the optimal performance and well-being of sport performers (American Psychological Association, 2019a), clinical psychology predominately focuses on the provision of health care to address psychological distress and promote well-being (American Psychological Association, 2019b). Opportunities for sport psychologists to work collaboratively with clinical psychologists, to share their knowledge and understanding of the issues, to provide the most appropriate psychological support for sport performers may be of particular value (Biggin et al., 2017). Sporting organizations, therefore, should acknowledge the importance of both sport and clinical psychology (Gorczynski et al., 2020), and utilize the novel findings of this study to holistically support sport performers’ psychological, social, and behavioral responses to high lifetime (non-sport and sport-specific) stressor exposure.

Given the increased interest in the mental health and well-being of sport performers (Gouttebarge et al., 2019), this study provides a timely investigation into sport performers’ experiences of high lifetime (non-sport and sport-specific) stressor exposure. A further strength was the use of creative and visual data collection methods (i.e., timelining), which encouraged a greater understanding of phenomena by prompting participants to reflect on their experiences in greater depth (Marshall, 2019). However, several limitations of this study should also be noted. First, the findings may only be specific to the participants sampled in this study. However, it is possible that the findings of this study may be generalized in certain ways. Specifically, naturalistic generalizability, in that the findings may resonate with sport performers who have had similar experiences to those in the present study (Smith, 2018). Second, the present study did not explore how sport performers’ responses to lifetime stressors (e.g., coping strategies) changed over time. Therefore, future research could use timelines to explore how lifetime stressor exposure shapes emotional and behavioral responses over time (Mazzetti & Blenkinsopp, 2012). Third, the findings of this study have been presented as discrete and separate themes. Despite this, however, it is important to recognize the potential interface between, and interactive impact of, the themes (i.e., cognitions, emotions, and behaviors; Arnold & Fletcher, 2012). Therefore, future research should attempt to explore this complexity. Finally, although this study was advertised equally to both genders, male sport performers were under-represented, possibly owing to the stigma that is associated with disclosing mental health problems among male athletes (Rice et al., 2021).

In conclusion, this qualitative study explored how relatively high levels of lifetime (non-sport and sport-specific) stressor exposure influenced sport performers’ health, well-being, and performance. We developed three overarching themes, highlighting the psychological (e.g., maladaptive coping strategies), social (e.g., difficulties in building and maintaining relationships), and behavioral (e.g., risky behaviors) factors. The in-depth experiences captured and subsequent themes identified extend theoretical and empirical knowledge and provide vital new information to help practitioners identify, and intervene with, sport performers who are at-risk of stress-related health, well-being, and performance problems.

## Supplementary Material

Supplementary Material
