# Supplementary figures and images for: A qualitative exploration of how lifetime stressor exposure influences sport performers’ health, well-being, and performance
Source: Anxiety Stress Coping. Author manuscript; Available in PMC 2024 Jul 1. (PMC11216060; doi:10.1080/10615806.2023.2246023)

# Timeline

## Example N° 1

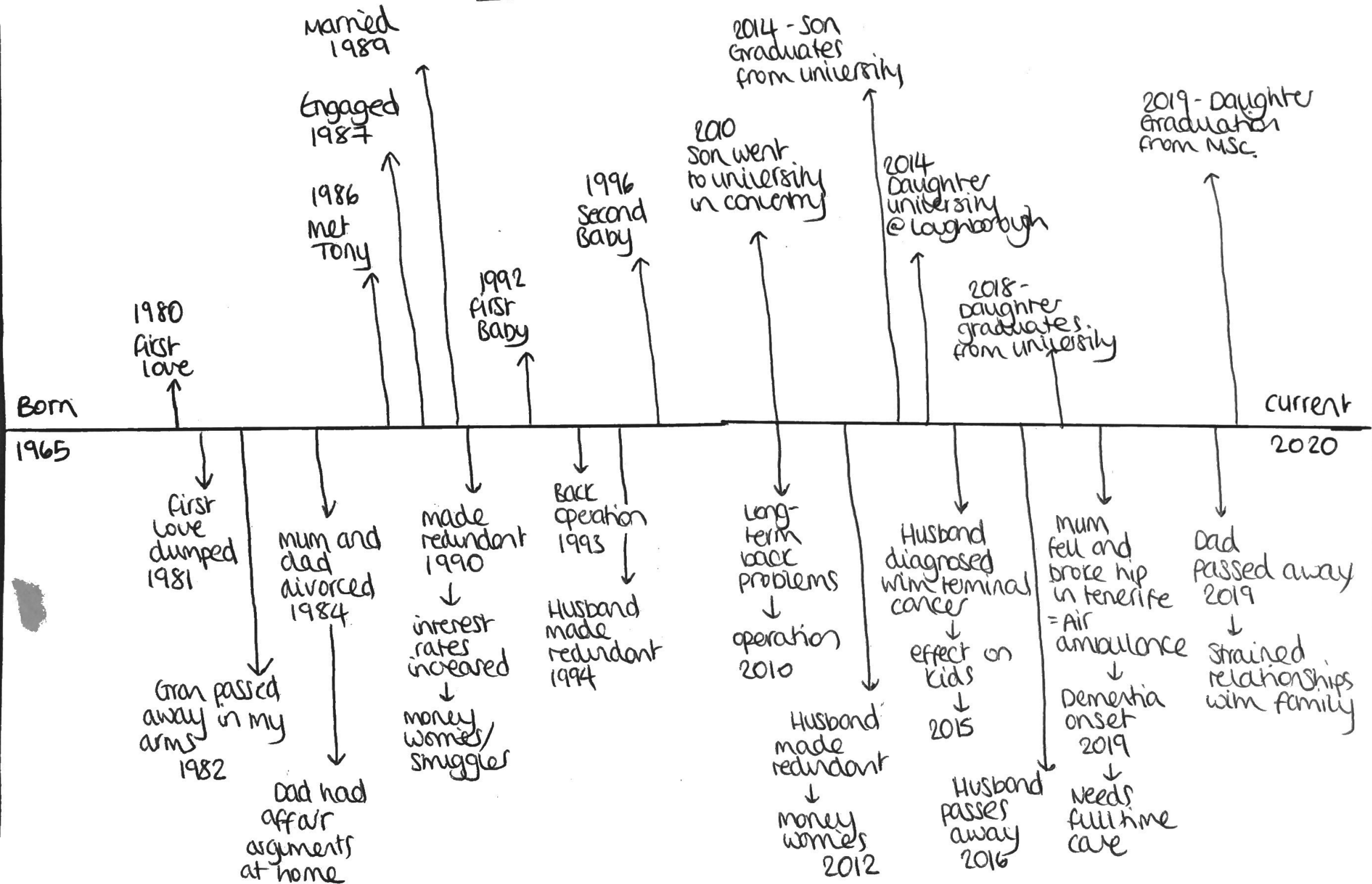

# My Timeline

Example N° 2

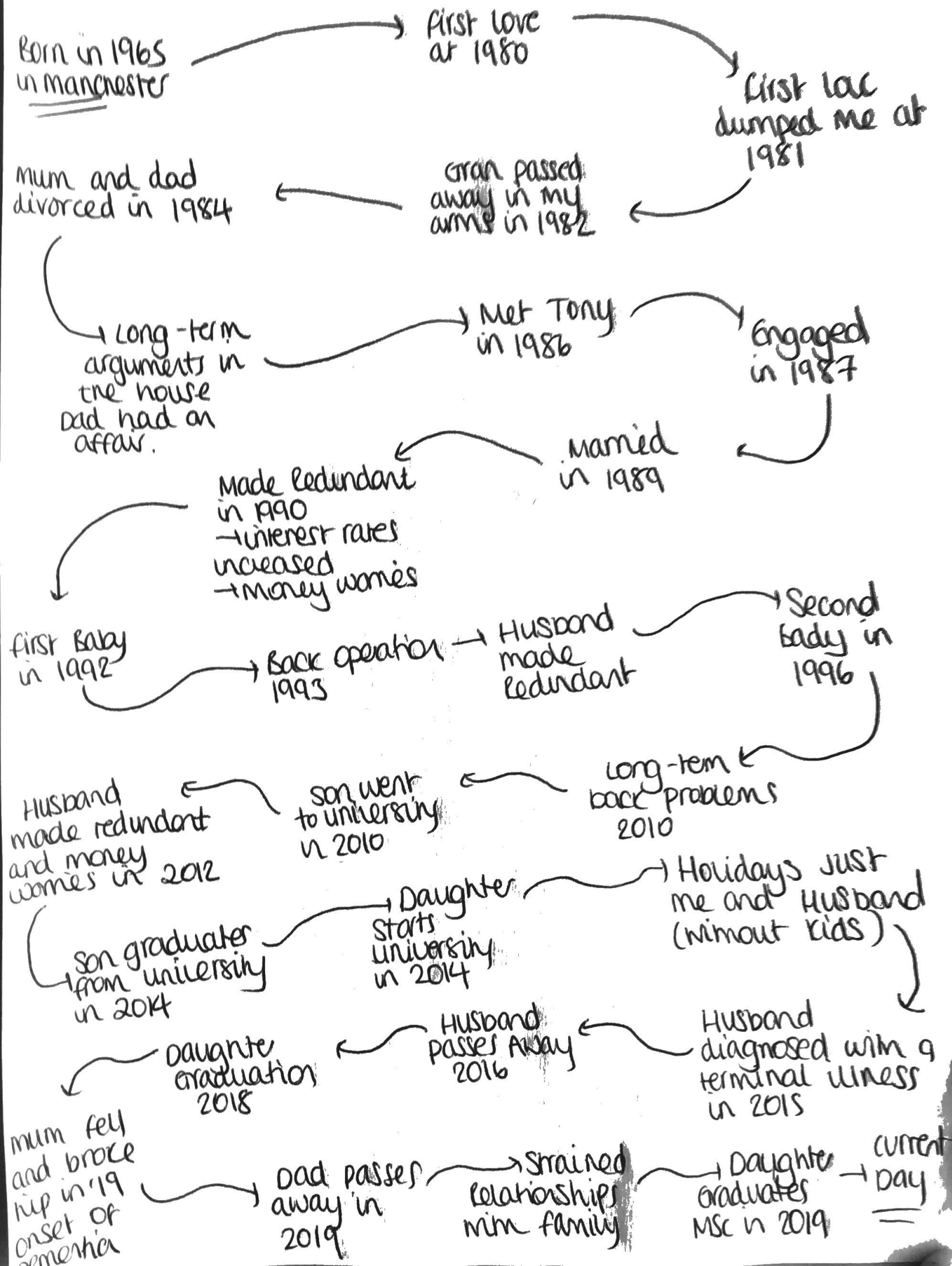

## Timeline

### Example N° 3

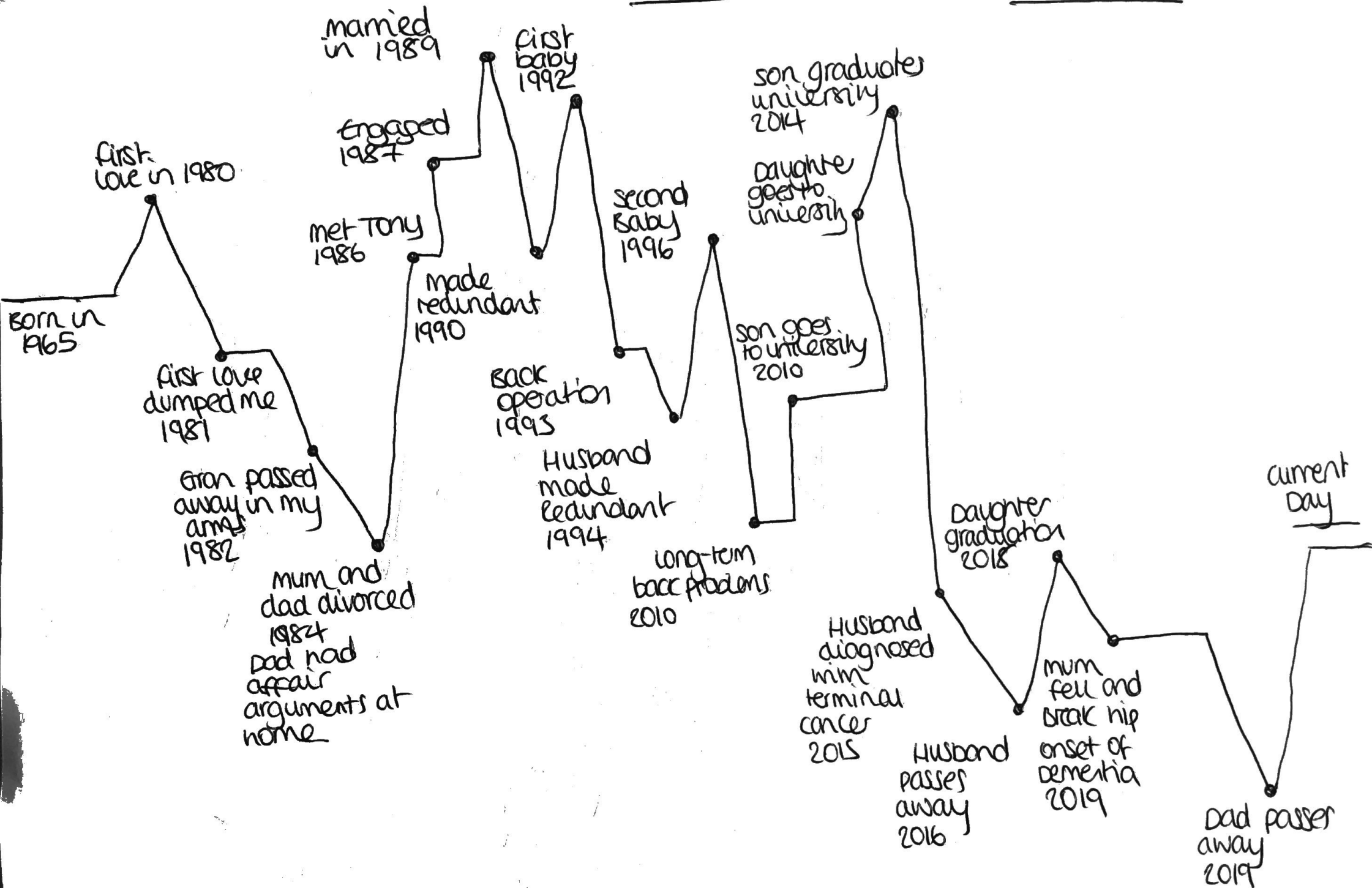

Supplement: Supplementary Material [file NIHMS2005056-supplement-Supplementary_Material.pdf]
